# A thrombophilia family with protein S deficiency due to protein translation disorders caused by a Leu607Ser heterozygous mutation in *PROS1*

**DOI:** 10.1186/s12959-021-00316-4

**Published:** 2021-09-08

**Authors:** Yan-ping Zhang, Bin Lin, Yuan-yuan Ji, Ya-nan Hu, Xin-fu Lin, Yi Tang, Jian-hui Zhang, Shao-jie Wu, Sen-lin Cai, Yan-feng Zhou, Ting Chen, Zhu-ting Fang, Jie-wei Luo

**Affiliations:** 1 Shengli Clinical Medical College of Fujian Medical University, Fuzhou, 350001 China; 2 Department of Interventional Radiology, Fujian Provincial Hospital, Fuzhou, 350001 China; 3 Department of Pediatrics, Fujian Provincial hospital, Fuzhou, 350001 China; 4 Department of Traditional Chinese Medicine, Fujian Provincial Hospital, Fuzhou, 350001 China

**Keywords:** Protein S, Deficiency, PROS1, Mutation, Vein thrombosis

## Abstract

**Background:**

Protein S deficiency (PSD) is an autosomal dominant hereditary disease. In 1984, familial PSD was reported to be prone to recurrent thrombosis. Follow-up studies have shown that heterozygous protein S (*PROS1*) mutations increase the risk of thrombosis. More than 300 *PROS1* mutations have been identified; among them, only a small number of mutations have been reported its possible mechanism to reduce plasma protein S (PS) levels. However, whether *PROS1* mutations affect protein structure and why it can induce PSD remains unknown.

**Methods:**

The clinical phenotypes of the members of a family with thrombosis were collected. Their PS activity was measured using the coagulation method, whereas their protein C and antithrombin III activities were measured using methods such as the chromogenic substrate method. The proband and her parents were screened for the responsible mutation using second-generation whole exon sequencing, and the members of the family were verified for suspected mutations using Sanger sequencing. Mutant and wild type plasmids were constructed and transfected into HEK293T cells to detect the mRNA and protein expression of *PROS1*.

**Results:**

In this family, the proband with venous thrombosis of both lower extremities, the proband’s mother with pulmonary embolism and venous thrombosis of both lower extremities, and the proband’s younger brother had significantly lower PS activity and carried a *PROS1* c. 1820 T > C:p.Leu607Ser heterozygous mutation (NM_000313.3). However, no such mutations were found in family members with normal PS activity. The PS expression in the cell lysate and supernatant of the Leu607Ser mutant cells decreased, while mRNA expression increased. Immunofluorescence localization showed that there was no significant difference in protein localization before and after mutation.

**Conclusions:**

The analysis of family phenotype, gene association, and cell function tests suggest that the *PROS1* Leu607Ser heterozygous mutation may be a pathogenic mutation. Serine substitution causes structural instability of the entire protein. These data indicate that impaired PS translation and synthesis or possible secretion impairment is the main pathogenesis of this family with hereditary PSD and thrombophilia.

## Background

Protein S (PS) is a vitamin K-dependent plasma glycoprotein that is mainly synthesized by hepatocytes or macrophages [1]. Forty percent of PS is free and has anticoagulant activity, while 60% of PS is bound to C4b and has no activity [2]. On the one hand, PS exerts an anticoagulant effect mainly by serving as a cofactor of activated protein C (APC) to promote the inactivation of factor V (FV) a and FVIIIa [3]. On the other hand, PS also serves as a cofactor of tissue factor pathway inhibitor (TFPI), which inhibits the activity of tissue factors by promoting the binding interaction of TFPI and FXa [4]. Hereditary protein S deficiency (PSD) is an autosomal dominant hereditary disease, which may be caused by genetic and acquired factors [5]. It is classified into three subtypes: Type I (total PS, free PS levels, and PS activity are decreased), type II (total PS and free PS levels are normal, but PS activity is decreased), and type III (total PS level is normal, but free PS level and PS activity are decreased) [6]. There is no significant difference among these three types of clinical manifestations, which are only identified by laboratory testing; 95% of patients with PSD develop type I and type III PSD [7].

As of September 6, 2021, there are more than 360 mutations in PSD-related genes in the Human Gene Mutation Database (HGMD) (http://www.hgmd.org). There are 276 types of missense/nonsense, 48 types of splicing, 4 types of regulatory, 54 types of small deletions, 25 types of small insertions, 6 types of small indels, 28 types of gross deletions, 7 types of gross insertions, as well as complex repeats that have not yet been identified. The most common causes of PSD are missense or nonsense substitutions, followed by splice site mutations, small or large repeats, insertions, or deletions [8]. The main clinical manifestations of most patients with heterozygous mutations in the protein S gene (*PROS1)* are lower extremity deep venous thrombosis and pulmonary embolism [9]. *PROS1* mutations are associated with an increased risk of venous thrombosis [10], and some reports suggest that *PROS1* variants increase the risk of arterial embolism, such as cerebral infarction and myocardial infarction [11]. About half of patients with PSD develop symptoms before the age of 55, while some of them have no complications for the rest of their lives [12]. In the past, we detected a new mutation in *PROS1* in a family prone to thrombosis, which had not been previously reported. In this study, we discuss the pathogenicity and pathogenesis of this mutation.

## Materials and methods

### Research subjects

The 16-year-old female proband (III5), of Han nationality, complained of “swelling and pain in the left lower limb for 3 days”. She was in good health and had no bad lifestyle-related habits, such as, smoking, drinking etc. Among the family members, her mother (II8) had a history of bilateral deep venous thrombosis of the lower extremities and pulmonary embolism, and her parents were from non-consanguineous marriages. Physical examination showed that the left lower limb of the proband had edema, especially on the dorsal foot, shank, and thigh. There were no obvious varicose veins, hyperpigmentation, skin ulceration, palpable nodules, or deep vein tenderness with a positive Homan’s sign. The circumference of both lower limbs was measured and was as follows: 15 cm above the left patella, 44 cm; 15 cm above the right patella, 39 cm; 15 cm below the left patella, 39 cm; and 15 cm below the right patella, 36 cm.

At the age of 39, II8 had complained of “distension and pain of the left lower limb for 2 days” in another hospital. She was in good health and had no special bad habits. Physical examination revealed swelling of the left lower limb. She was diagnosed with “deep venous thrombosis of the left lower limb” and was treated with anticoagulation and thrombolysis. After that, she improved and was discharged from the hospital and took anticoagulants regularly for a long time. A year ago, she visited the hospital again due to “sudden chest pain with loss of consciousness” and was diagnosed with “pulmonary embolism.” .

## Methods

### Clinical phenotype detection

Clinical phenotypes and clinical biochemical indicators were collected from the proband and her related family members. Clinical biochemical indexes included PS activity, as measured using the coagulation method. Its principle is that protein C can hydrolyze coagulation factors VA and VIIIa in the coagulation waterfall reaction activated by RVV. The extension of coagulation time can reflect the activity of PS in the sample. The coagulation time of patients can calculate the content of free PS (FPS) from the standard curve. The sample must be centrifuged twice to remove platelets from the plasma sample (platelets in the plasma should be less than 10 × 109 / L) and frozen for inspection. The operation was performed according to the protein S kit instructions and completed by KingMed Diagnostics (Guangzhou, China). The activity of protein C (PC) and antithrombin III (AT), as measured using the chromogenic substrate method, blood routine, coagulation function, and biochemistry. FPS: Ag and TPS: Ag were used ELISA method (KingMed Diagnostics, Guangzhou, China) to measure.

### Extraction of genomic DNA

Peripheral blood (8 mL) of the proband and peripheral blood (2 mL) of each family member were collected in ethylenediaminetetraacetic acid anticoagulant tubes, and genomic DNA of the proband and her family members was extracted using the QIAGEN DNA Blood Mini Kit (Cat# 51106, QIAGEN Co. Ltd., Shanghai, China).

### Location and screening strategy of mutant genes

The TargetSeq® liquid probe hybridization and capture technique independently developed by Igen iGeneTech® (Beijing, China) was used to establish a genomic DNA library and capture the promoter and exon regions (16.06 Mbp) of 5,081 genes related to genetic diseases. Paired end 150 bp sequencing was performed using the Illumina X10 or NovaSeq 6000 platform. The captured target genes were *PROS1* and Serpin family C member 1 (*SERPINC1*). Based on the results of BAM alignment with the genome reference sequence, single-nucleotide variants and indels in the samtools, GATK, and ANNOVAR sequencing results were used to remove the variation sites with intermediate frequency higher than 0.01 in ExAC, gnomAD, iGeneTechDB (local database with more than 10,000 samples), benign and likely benign mutations in ClinVar, and synonymous_variant mutations in the Human Genome Variation Society. Combined with the exon sequencing data of the parents, the sources of mutation were annotated and divided into three types: those from the father, from the mother, and suspected to be new mutations. The Hemostasis Thrombosis Expert Panel of the OMIM Phenotypic Series-PS188050 and CLINGENE were used to search for genes. Mutations from the father were excluded (the mutations from the mother and the suspected new mutations were retained), and two mutations in *SERPINC1* and *PROS1* were obtained. Sorting Intolerant from Tolerant (SIFT, http://sift.jcvi.org/), Polymorphism Phenotyping (PolyPhen-2, http://genetics.Bwh.harvard.edu/ppH2/) and Mutation Taster (http://mutationtaster.org/) were used to predict the pathogenicity and harmfulness of the mutations. The upper and downstream positions of the sequence of the target mutation site were designed using Premier 5.0, and the target area was amplified. The corresponding suspected pathogenic mutations were verified by Sanger sequencing using the ABI3500Dx platform. The amplified fragment length of c. 1820 T > C:p, the Leu607Ser sequence of the mutation point in *PROS1* (NM_000313.3), was 498 bp. The primers F: CTGGCTGGGATAGCCAAATGA and R: CTTGCTTATATTGAATCTTTGCTCTGC were used for amplification (melting temperature, 62.5 °C). The amplified fragment length of c.883G > A:p, the Val295Met sequence of *SERPINC1* (NM_000488.3), was 407 bp. The primers F: CTTGCAGCTGCTCCTTCAAACT and R: TGTCTTGTGTCAATAACTATCCTCCTA were used for amplification (melting temperature, 61 °C). Synbio Technologies Co., Ltd. (Suzhou, China) synthesized all primers.

### Construction and identification of *PROS1* wild type (WT) and p.Leu607Ser mutant plasmids

The plasmid synthesis scheme pcDNA3.1–3 × Flag-C was used as the expression vector to synthesize *PROS1* with a KpnI/XhoI cleavage site. The WT plasmid 1 (pcDNA 3.1-PROS1WT-3 × Flag-C) and the mutant plasmid 2 (pcDNA3.1-PROS1mut-3 × Flag-C) were constructed, both with a KpnI/XhoI restriction enzyme site. The mutant plasmid 2 contained the 1820 T > C mutation in *PROS1*. Target genes were amplified and sequenced. The cloning of *PROS1* (WT) and *PROS1* (1820 T > C) and the synthesis of related polymerase chain reaction (PCR) primers were performed by Wuhan Gene Create Biological Engineering Co. Ltd.(Wuhan, China).

### Cell transfection

HEK293T cells were digested and collected using trypsin, and the cells were placed into a 10 cm petri dish at a density of 1–2 × 10^7^ cells/plate in an appropriate complete culture medium. After adhesion, the total area of the cells reached 80–90% confluence. According to the conditions of cell adhesion, cells were incubated at 37 °C in an incubator containing 5% CO_2_ for 8–24 h, and transient transfection was started after the cells were completely adhered. According to the instructions for TurboFect (R0531, Thermo, Massachusetts, USA), TurboFect-DNA Mix was prepared and mixed with DNA plasmids (10 μg/*PROS1* WT, mutant, or control plasmid + 5 μg green fluorescent protein [GFP]) and 30 μL TurboFect in 1000 μL Opti-Medium. After incubation at room temperature for 15 min, TurboFect-DNA Mix was added to the petri dish. After 12 h, the complete medium was changed, and HEK293T cells were cultured for 48 h. Cells were observed to be in good condition by microscopy and the culture medium was collected for further evaluation.

### Quantitative real-time (qRT)-PCR detection

HEK293T total RNA was extracted according to the TriPure Isolation Reagent kit (11,667,165,001, Roche, Shanghai, China), and the difference in the *PROS1* transcription levels was detected by reverse transcription and qRT-PCR. The first chain of cDNA was synthesized according to HiFiScript (CW2020M, CWBIO, Beijing, China). The reaction system contained 2.5 mM dNTP Mix, 4 μL; primer mix, 2 μL (primers in Table 1); RNA Template, 7 μL; 5× RT Buffer, 4 μL; 1× dithiothreitol, 0.1 M, 2 μL; 10 mM HiFiScript, 200 U/μL; and RNase-free water, 20 μL. After mixing the liquid using a vortexer, the tube was centrifuged for a short time. The product was incubated at 42 °C for 50 min and at 85 °C for 5 min. The cDNA obtained by reverse transcription was diluted 20-fold, and 40 RT-qPCR cycles were performed in a Roche LightCycler 480 (Roche, Beijing, China).

**Table 1:** Primers for qRT-PCR

hPROS1 qRT F	CCCGGAAACGGATTATTTTT
hPROS1 qRT R	CTCCTTGCCAACCTGGTTTA
hGAPDH F	AGAAGGCTGGGGCTCATTTG
hGAPDH R	AGGGGCCATCCACAGTCTTC
CopGFP qRT F	AGGACAGCGTGATCTTCACC
CopGFP qRT R	CTTGAAGTGCATGTGGCTGT

### Western blot detection

HEK293T cells were cultured, lysed, total protein was extracted, and PROS1 expression was detected. Protein samples were separated using electrophoresis and then wet transferred to a polyvinylidene fluoride (PVDF) membrane, soaked in 5% skim milk prepared in Tris-buffered saline with 0.1% Tween® 20 (TBST), and sealed at room temperature for 1 h. Next, the membrane was washed once and anti-protein S antibody (97,387, Abcam, UK, 1: 500) or actin antibody (ab8227, Abcam, UK, 1: 5000) was added. The Flag antibody (F3165, Sigma, USA, 1:500), diluted with 5% bovine serum albumin (BSA), was added to the membrane overnight at 4 °C, and the membrane was washed thrice. Horseradish peroxidase-labeled secondary antibodies (goat anti-rabbit IgG, 1:2000 or goat anti-mouse IgG 1:2000, diluted with 5% BSA, ab6721 and ab6789, respectively, Abcam, UK) were added to the membrane and then incubated in a shaker at room temperature for 1 h. The PVDF membrane was washed with TBST five times and with ddH_2_O once before exposure.

### Enzyme linked immunosorbent assay (ELISA) of PROS1 in HEK293T cell lysates and cell supernatants

According to the instructions of the Human Protein S ELISA Kit (ab190808, Abcam, UK), the working standard liquid was prepared, and PROS1 expression in HEK293T cell lysates and cell supernatant was detected. A microplate reader (Varioskan Lux, Thermo, Massachusetts, USA) was used to measure the optical density at 450 nm immediately after the substrate solution was added to stop the reaction. A standard curve was created and PROS1 levels in the sample were calculated.

### Immunofluorescence localization experiment

After being fixed, permeabilized, and blocked, the transfected HEK293T cells were incubated at 4 °C overnight with the PROS1 primary antibody (diluted 1:200). The transfected HEK293T cells were rinsed with phosphate-buffered saline (PBS) thrice, the fluorescent secondary antibody (diluted 1:500) was added and incubated at room temperature in the dark for 2 h, rinsed with PBS thrice, and stained with 4′,6-diamidino-2-phenylindole. The transfected HEK293T cells were incubated at room temperature for 5 min and rinsed twice with 1× PBS for 3 min each time. A laser confocal microscope (Nikon A1, Shanghai, China) was used to capture images.

### Statistics

Experimental data were statistically analyzed using GraphPad Prism 6.02. An unpaired *t*-test was used to compare the two groups. The mean value was expressed as the mean ± standard error of the mean (SEM), and *p* < 0.05 indicated that the difference was statistically significant.

## Results

### Clinical phenotypes

**Fig. 1:**
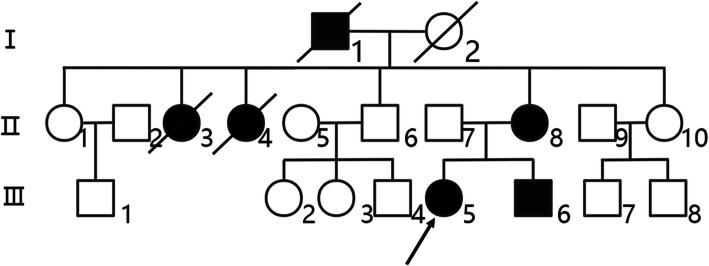
Family map. In the family map of hereditary protein S deficiency, the proband (III5) and other patients carry a c. 1820T > C (p.Leu607Ser) *PROS1* heterozygous mutation

The 16-year-old proband (III5) was examined for blood coagulation function (Table 2). The indices related to blood coagulation had no significant changes. Color Doppler ultrasound of the lower limb vein showed thrombosis of the left external iliac vein and deep vein of the left lower limb. Digital subtraction angiography showed the distal left superficial femoral vein and the left inferior vena cava had thrombosis. PS activity, total protein S (TPS) and free protein S (FPS) was significantly decreased, while the PC and AT activities were normal; thus, it was considered asthrombosis caused by type I PSD. The mother of the proband (II8), 42 years old, had a history of recurrent venous thrombosis. For the first time, when lower limb swelling and pain occurred, Color Doppler ultrasound and Computed tomographic angiography (CTA) indicated thrombosis of the left lower extremities. The second time, when chest pain occurred, CTA showed pulmonary embolism. Color Doppler ultrasound showed that the deep vein of the left lower extremity and the right popliteal vein were partly recanalized after thrombosis. PS activity, TPS and FPS was significantly decreased and the activity of AT and PC was normal, so it was suspected that type I PSD caused thrombosis in II8 many times. The 13-year-old younger brother of the proband (III6) had no history of thrombosis. His PS activity, TPS and FPS was significantly decreased and the PC and AT activities were normal; thus, he was diagnosed with typeIPSD. The father of II8 (I1) at the age of 63 died of “pulmonary embolism”. The older sister of II8 (II3), 45 years old, died of “pulmonary embolism”. Another older sister (II4) died of “pulmonary embolism”,when she was 49 years old. The father of III5 (II7), 43 years old, was in good health. To date, no thrombosis has been found in other family members (II1, II10, II6, III1, III2, III3, III4, III7, III8). Blood coagulation function of all members are displayed in the Table 2. A pedigree map of the genetic family was drawn (Fig. 1).

**Table 2:** Coagulation function indexs of proband and family members in hereditary protein S deficiency family

Items	Propositus (III5)	Father (II7)	Mother (II8)	Brother (III6)	Member (II1)	Member (II6)	Member (II10)	reference value
PT (s)	13.3	11.2	11.8	12.3	9.8	11.9	12.1	9.9–12.9
APTT (s)	27.9	26.4	28.8	32	27.1	24.3	23.9	23.3–32.5
TT (s)	15.5	15.8	17.0	30.9	17.1	16.4	19.2	14–21
Fg (g/L)	2.57	2.32	2.7	1.9	2.41	2.79	2.34	1.8–3.5
D-dimer	25.8	0.24	13.2	15.5	0.23	0.01	0.01	0–0.55
TPS (mg/L)	67	208	85	71	–	–	–	160–260
FPS (mg/L)	19	66	22	23	–	–	–	48–67
PS(%)	< 16	84.4	< 16	16.7	102.7	97.4	112.3	Male:75–130 Female:52–118
PC(%)	66.9	90.1	100.2	60.2	113.4	124.2	98.1	70–140
AT-III(%)	92	107.6	97.4	85.9	121.2	109.1	97.1	75–125

Note: *PT* prothrombin time; *INR* international normalized ratio; *APTT* activated partial thromboplastin time; *TT* thrombin time; *Fg* fibrinogen; *FDP* fibrin degradation products; *TPS* total protein S; *FPS* free protein S; *PS* protein S; *PC* protein C; *AT* antithrombin III

### Screening for a thrombophilia gene mutation

The members of the family were analyzed by whole exon sequencing, and two mutation sites were identified in proband III5. One was a heterozygous mutation of exon 14 in *PROS1* (NM_000313.3): c.1820 T > C(p.Leu607Ser); the transformation from TTG to TCG was not recorded in the ClinVar and HGMD databases. Thus, this is a newly discovered mutation, and its pathogenicity is unclear. The other was a heterozygous mutation of exon 3 in *SERPINC1* (NM_000488.3): c.883G > A (p.Val295Met) (rs201381904). This mutation has not been recorded in the ClinVar and HGMD databases, and its pathogenicity is not clear, but the AT plasma levels in the members of this family were normal, which ruled out the diagnosis of hereditary AT deficiency. According to SIFT [13] and PolyPhen-2 [14] scores, the *PROS1* mutation SIFT score is 0, PolyPhen-2 score is 1, and Mutation Tester [15] predicts that protein function is moderately affected. The *SERPINC1* mutation SIFT score was 0.036, and the PolyPhen-2 score was 0.996. The lower the SIFT score, the greater the harm and the closer the PolyPhen-2 score to 1, the stronger the pathogenicity. The *PROS1* L607S heterozygous mutation and *SERPINC1* V295M heterozygous mutation were identified in II8, and a *PROS1* L607S heterozygous mutation was also identified in III6. Except for the heterozygous mutation V295M carried by II1, there were no L607S and V295M mutations in other family members.

### Cloning of *PROS1* WT and p.Leu607Ser gene mutations

The *PROS1* WT and *PROS1*/p.Leu607Ser cloning and eukaryotic expression vectors were successfully constructed. Fragments of WT *PROS1* and mutant *PROS1*/p.Leu607Ser digested by KpnI/XhoI were approximately 1138 bp, which was consistent with plasmid design. The constructed vectors were verified by sequencing and transfected successfully into HEK293T cells.

### Localization of WT PROS1 and its mutants in cells

**Fig. 2:**
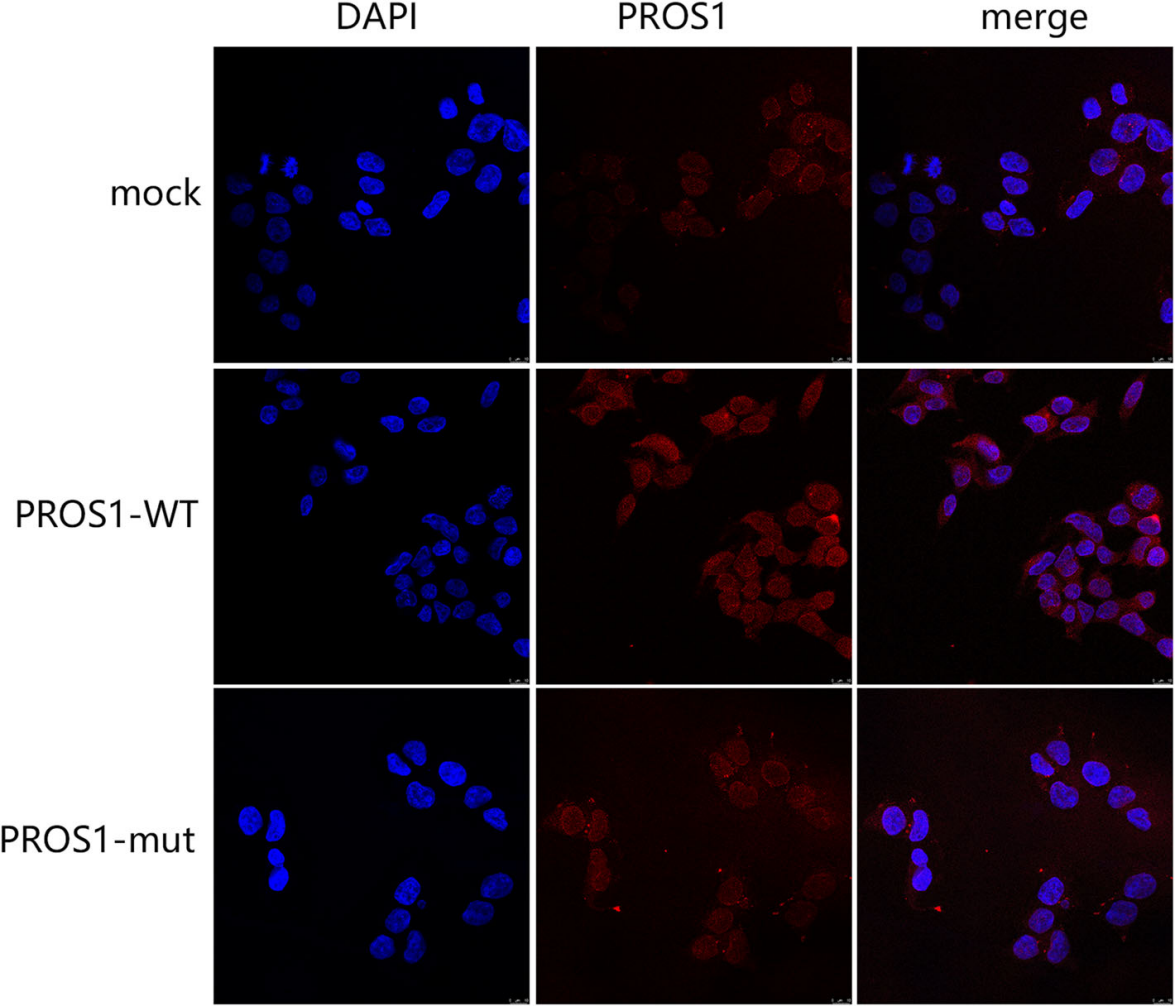
Localization of protein S (PROS1). Localization of PROS1 mock, wild type (*PROS1*-WT), and the p.Leu607Ser mutation (*PROS1*-M) in HEK293T cells, as detected by immunofluorescence. There is no difference before and after introduction of the *PROS1* 1820T > C mutation. PROS1 expression was obviously downregulated after mutation

The localization of WT and mutant PROS1 was detected by immunofluorescence (Fig. 2), which showed that PROS1 was distributed in the cytoplasm of the cells. There was no significant difference in intracellular fluorescence localization before and after introduction of the *PROS1* 1820 T > C mutation. However, the expression intensity of PROS1 protein after mutation decreased significantly, considering the reduction of PROS1 protein synthesis due to mutation.

### Expression of WT *PROS1* and its mutants in HEK293T cells

**Fig. 3:**
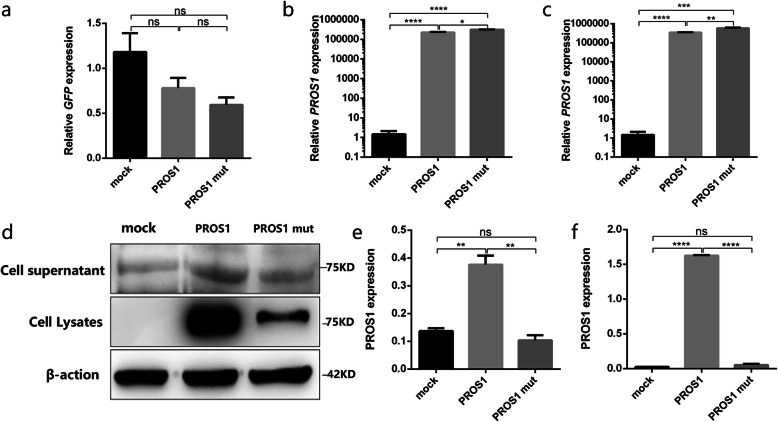
Relative protein S (PROS1) mRNA and protein expression. (**a**) Relative green fluorescent protein (GFP) expression of cells transfected with mock, wild type (*PROS1*-WT), and the p.Leu607Ser mutation (*PROS1*-M) shows that there is no significant difference in transfection efficiency between the three groups. (**b**) Relative *PROS1* mRNA expression of mock, *PROS1*-WT, and *PROS1*-M in HEK293T cells with glyceraldehyde-3-phosphate dehydrogenase (GAPDH) as an internal reference. (**c**) Relative *PROS1* mRNA expression of mock, *PROS1*-WT, and *PROS1*-M in HEK293T cells using GFP as an external reference. (**d**) PROS1 expression of mock, *PROS1*-WT, and *PROS1*-M in the supernatant and lysate of HEK293T cells, as detected by western blot. (**e**) According to the enzyme-linked immunosorbent assay (ELISA) standard curve, the expression of PROS1 mock, *PROS1*-WT, and *PROS1*-M in the supernatant of HEK293T cells is calculated. (**f**) According to the ELISA standard curve, the expression of PROS1 mock, *PROS1*-WT, and *PROS1*-M in HEK293T cell lysate is calculated. *p<0.05,** *p*<0.01,*** *p*<0.005, *n* = 3

The relative mRNA expression of the WT (*PROS1*-WT) and p.Leu607Ser mutant (*PROS1*-MUT) *PROS1* in HEK293T cells was detected using qRT-PCR. The difference between the *PROS1* mRNA expression groups was compared to that of actin (Fig. 3b) as an internal reference when there was no significant difference in transfection efficiency among groups (Fig. 3a). *PROS1* mRNA expression was significantly upregulated with the *PROS1* 1820 T > C mutation (*p* < 0.05). GFP was used as an external reference (Fig. 3c) to compare the difference between the *PROS1* mRNA expression between groups. Again *PROS1* mRNA expression was significantly upregulated with the *PROS1* 1820 T > C mutation (*p* < 0.01). PS expression (Fig. 3d) in the cell supernatant and lysate was detected by western blotting. PROS1 expression was significantly downregulated with the mutation. At the same time, PROS1 expression in the supernatant of the cell culture medium and cell lysate was detected using ELISA (Fig. 3e, f). PROS1 expression levels in the culture medium supernatant and cell lysate of the p.Leu607Ser mutant group was significantly lower than that of the WT group, which was consistent with the results of western blotting.

### Protein of bioinformatics prediction

**Fig. 4:**
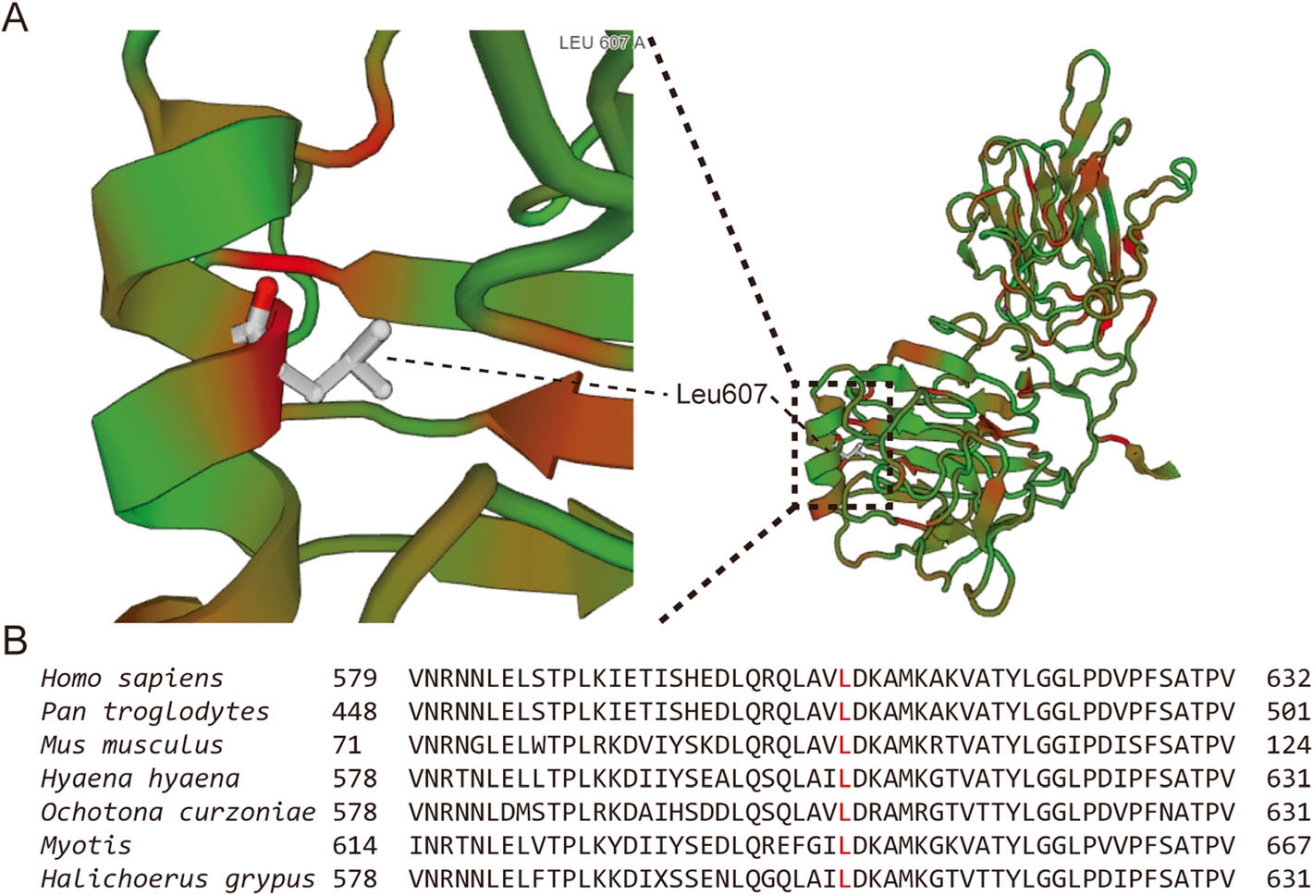
Protein S (PROS1) homology modeling and analysis. **a** Homology modeling of PROS1 has been performed using Swiss-Model. Leu607 is labeled in the alpha helix. **b** Conserved analysis of amino acid sequences near Leu607 (marked with red) (https://swissmodel.expasy.org/repository/uniprot/P07225?template=1h30.1.A&range=266-673)

**Fig. 5:**
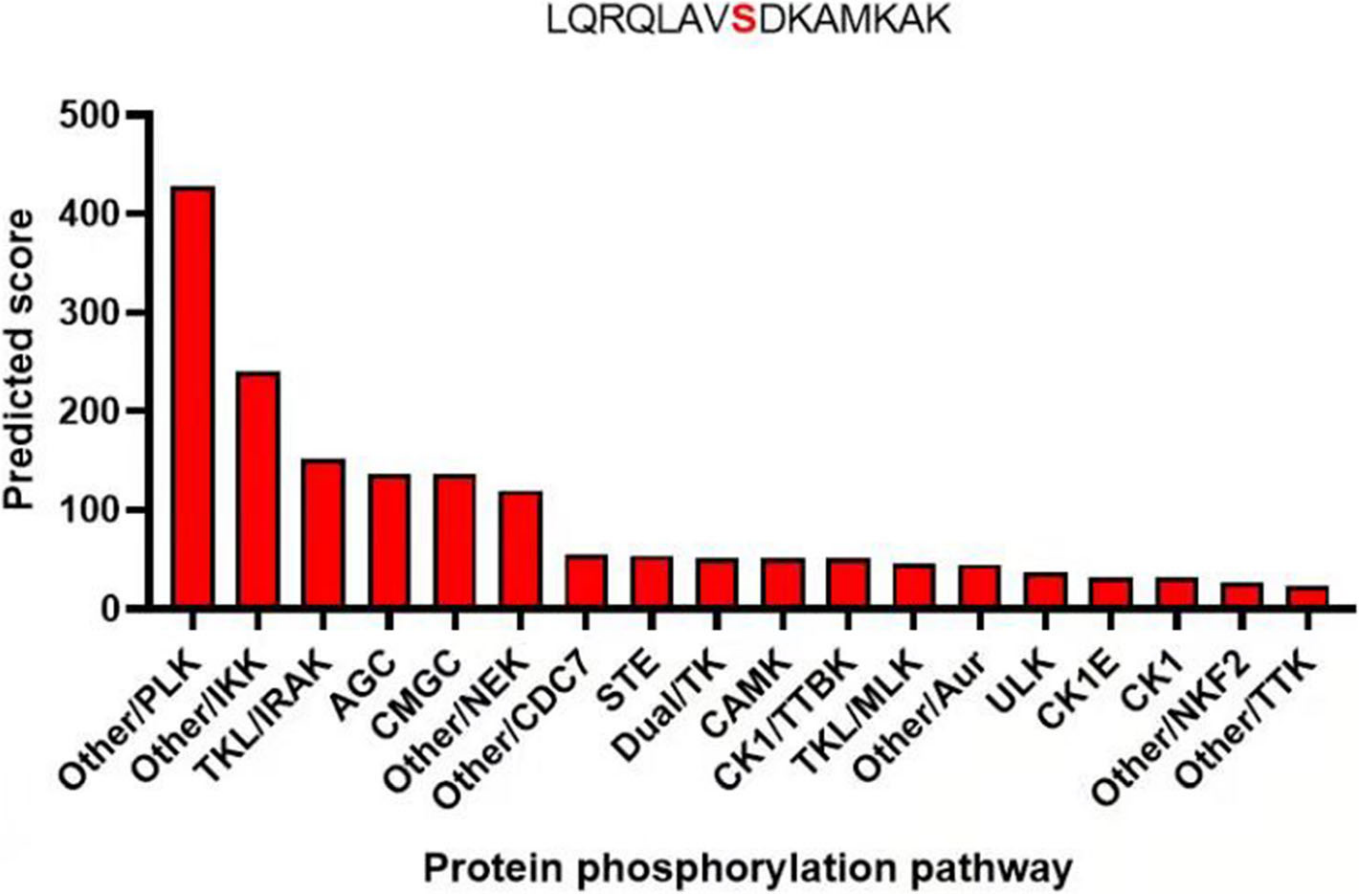
Prediction of protein phosphorylation pathway on Leu607Ser using GPS 5.0 software ranked by possibility

The mutant (https://swissmodel.expasy.org/interactive/7J8pZH/models/) and WT PROS1 (https://swissmodel.expasy.org/interactive/HxBw4h/models/) homologous proteins were constructed using the Swiss Model (Fig. 4a). The characteristics of the advanced structure were observedl. The number of amino acid residues in the Ca^2+^ region of the mutant protein was one less than that of the WT protein. In humans and other mammals, a comparison between Leu607 of PS and the adjacent flanking structures of PS show that this site is highly conserved (Fig. 4b). Prediction of protein phosphorylation pathway on Leu607Ser using GPS 5.0 software (http://gps.biocuckoo.org/index.php). The results show that L607S is more likely to pass through polo-like kinase (PLK) pathway (Fig. 5).

## Discussion

In this study, we found a new mutation site *PROS1* c. 1820 T > C (p.Leu607Ser) for the first time. In previous studies a missense *PROS1* mutation (Gly222Arg) has been identified in a patient with pulmonary embolism, which causes PS activity to decrease to 5.0% [16]. The PS activity of several codon mutations near L607, such as Ser627fs, Ser627 ins101fsX34 (acc HGMD nomenclature), p.Ala536Val, p.Asn583His, p.Thr617Ile, p.Asp624His, and p.Cys666Ser (acc HGVS nomenclature) are all less than 40%, and the lowest is 12%, suggesting that mutation of the corresponding domain causes serious functional defects [17]. *PROS1* is located near the centromere of chromosome 3 (3q11.1); it contains 15 exons and encodes PS [18]. From the N-terminal to the C-terminal, there is a γ-carboxyl glutamate domain, a region sensitive to cleavage by thrombin, four domains homologous to epidermal growth factor, and a region homologous to sex hormone binding globulin (SHBG). SHBG contains two tandem laminin G regions (LG1 and LG2) [19]. Mutations in *PROS1* are a risk factor for thrombosis in Asian populations and repeated spontaneous DVT and pulmonary embolism without obvious reasons are the most common symptoms [20]. Whether individuals with *PROS1* mutations have thrombosis greatly depends on the interaction between genes and the interaction between genes and the environment. However, compared to individuals without gene mutations, the risk of thrombosis with gene mutations is 2–11 times higher [21].

Although not every patient with PSD has a clinical phenotype, it will obviously increase the risk of thrombosis, especially in patients with heterozygous mutations and PS activity less than 30% [22]. The occurrence of clinical phenotypes is related to age, sex, and mutation type [23]. There are more male patients with hereditary PSD than female patients with hereditary PSD, but the peak age in females is younger, which is due to the influence of hormones and risk factors, such as trauma, surgery, and oral contraceptives [24]. If *PROS1* occurs as a homozygous mutation, the prognosis is poor and the child may die of fulminant purple spot caused by severe PSD in the neonatal period [25]. Similar to the results of an animal experiment, explosive bleeding in *PROS1*^−/−^homozygous mice is observed in mice with *PROS1* knockout at the embryonic stage. *PROS1*^-/+^heterozygous mice survive to adulthood, but different degrees of vascular injury and dysplasia are observed [26]. At the same time, the level of PROS1 and the activity of auxiliary APC are detected, which are significantly lower than those of WT mice [26].

*PROS1* L607S may be because the mutated residues affect the level of translation and post-translation modification, resulting in disordered protein processing and secretion, which is the main molecular disease mechanism of most missense and other mutations in genetic diseases [27]. Conformation of SHBG and may be important in the PS anticoagulant effect; about half of the *PROS1* mutations in the LamG domain involve the acquisition and loss of residues with unique physical and chemical properties, such as cysteine, proline, and glycine, which directly affect PS function [28]. Because most of the mutant residues are hydrophobic, changes in these residues may affect protein folding and secretion [29]. The PS level and activity of D38Y and P626L mutants is significantly decreased in transfected COS-7 cells [30]. In this study, the hydrophobic amino acid Leu at position 607 was replaced by the polar neutral amino acid Ser, which is easily phosphorylated by protein kinase. Phosphorylation is the most important post-translational modification and has the greatest effect on the local and overall structural changes in proteins; most phosphorylation occurs on serine residues [31]. The phosphate group formed after phosphorylation of the mutated Ser607 may form hydrogen bonds or salt bridges with the adjacent Lys609, making the local structure compact, which may change the overall conformation of the protein and the interaction between proteins to regulate function [32]. In the mutant protein structure, Ser607 phosphorylation may also affect the binding force of calcium binding region. Ca^2+^ regulates the binding of the C-terminal SHBG region of bound PS to C4 binding protein (C4BP) [33]. Both LG1 and LG2 are involved in PS binding to C4BP, showing anticoagulant activity independent of APC [34]. If PS residues Lys423, Lys427, and Lys429 are replaced by other polar amino acids, the binding force between PS and C4BP is reduced by 5–10-fold. Insertion of alanine at position 611 leads to the loss of binding to C4BP [35], which leads to a decrease in anticoagulant function. The anticoagulant activity of free PS through the tissue factor pathway inhibitor (TFPI) is also through the combination of SHBG and TFPI, which further promotes the interaction between TPFI and FX a, thus inhibiting the activity of tissue factor [2]. LG1 and LG2 are necessary for the binding of SHBG and TFPI, but LG1 plays a major role [36]. A R474C mutant in LG1 reduces PS secretion by eight-fold and shortens the half-life of radioactive markers in transfected cells [37]. Because R474C mutation may lead to PS secretion disorder and intracellular degradation [37]. Then, the mechanism of endoplasmic reticulum-associated protein degradation is initiated, which leads to the decomposition of related proteins in cells [38].

In recent years, the relatively new drug is Novel oral anticoagulants (NOAC). NOACs is a highly selective anticoagulant, including factor Xa inhibitor rivaroxaban and direct thrombin inhibitor dabigatran. NOACs drugs have been shown to be more effective and safer than warfarin in randomized controlled trials, which can significantly reduce the incidence of stroke and thrombotic events [39]. Its therapeutic effect in patients with severe inherited thrombophilia is unclear, although there was one case of good benefit after using dabigatran [40].A recent report indicated patients with severe inherited thrombophilia had a good effect after receiving NOACs, but it was less effective in patients with PSD [41].

However, this research also has some limitations that only using bioinformatics analysis software to predict the possible effect of mutation sites on the structure of PROS1 protein, lacking experimental evidence, so we can further study the influence of 607Ser on protein. The specific mechanism remains undefined, and the mutation site affects which step in the secretion process, which needs to be further investigated.

## Conclusions

In this study, a heterozygous mutation of *PROS1* c.1820 T > C:p.Leu607Ser, was identified as a pathogenic mutation that caused disorderly PS translation, synthesis, and secretion or intracellular degradation, and finally led to a decrease in PS levels and activity, resulting in type I PSD. Heterozygous mutation of *PROS1* c.1820 T > C:p.Leu607Ser was familial.

## Data Availability

The datasets used and/or analyzed during the present study are available from the corresponding author upon reasonable request.

## Abbreviations

PS: protein S
PROS1: protein S gene
PSD: protein S deficiency
SERPINC1: Serpin family C member 1
TFPI: tissue factor pathway inhibitor
APC: activated protein C
qRT-PCR: quantitative real-time-polymerase chain reaction
DVT: deep vein thrombosis
PC: protein C
AT: antithrombin III
WT: wild type
PVDF: polyvinylidene fluoride
BSA: bovine serum albumin
ELISA: Enzyme linked immunosorbent assay
PBS: phosphate-buffered saline
PT: prothrombin time
INR: international normalized ratio
FDP: fibrinogen degradation product
TT: thrombin time
CDFI: Color Doppler flow imaging
CTA: Computed tomographic angiography
SHBG: sex hormone binding globulin
LG: laminin G regions
C4BP: C4 binding protein
NOAC: Novel oral anticoagulants
